# Expansion of a multi-pronged safe sleep quality improvement initiative to three children’s hospital campuses

**DOI:** 10.1186/s40621-020-00256-z

**Published:** 2020-06-12

**Authors:** Traci Leong, Kerryn Roome, Terri Miller, Olivia Gorbatkin, Lori Singleton, Maneesha Agarwal, Sarah Gard Lazarus

**Affiliations:** 1 Department of Biostatistics and Bioinformatics, Emory University Rollins School of Public Health, Atlanta, 30322 USA; 2 Emory University School of Medicine, Atlanta, 30322 USA; 3 Georgia Department of Public Health, Atlanta, 30303 USA; 4 Children’s Healthcare of Atlanta, Atlanta, 30322 USA; 5 Morehouse School of Medicine, Atlanta, 30310 USA; 6 Pediatric Emergency Medicine Associates, Atlanta, 30342 USA

**Keywords:** Infant mortality, Sleep safety, Sudden unexplained infant death, Quality improvement

## Abstract

**Background:**

The American Academy of Pediatrics (AAP) recommends infants should be **A**lone, on their **B**ack, and in a clear **C**rib to combat relatively stagnant rates of sudden unexpected infant death (SUID). These are referred to as the ABCs of safe sleep. Studies have shown these recommendations are not consistently followed in the hospital setting, but further investigation would determine how to improve the rate of adherence. The objective of this study was to evaluate the impact of an expanded safe sleep initiative at three Georgia free-standing children’s hospital campuses before and after a multipronged safe sleep initiative.

**Methods:**

A quality improvement program with a pre/post analysis was performed using a convenience method of sampling. Infants < 12 months old in three inpatient pediatric campuses were analyzed pre- and post- interventions. The intervention included: 1) nursing education, 2) identification of nurse “safe sleep” champions, 3) crib cards, 4) crib audits, and 5) weekly reporting of data showing nursing unit ABC compliance via tracking boards. The goal was ABC compliance of ≥25% for the post-intervention period. A standardized crib audit tool evaluated sleep position/location, sleep environment, and ABC compliance (both safe position/location and environment). Chi square analysis, Fisher’s exact test, and logistic regression were used to compare safe sleep behaviors before and after the interventions.

**Results:**

There were 204 cribs included pre-intervention and 274 cribs post-intervention. Overall, there was not a significant change in sleep position/location (78.4 to 76.6%, *p* = 0.64). There was a significant increase in the percent of infants sleeping in a safe sleep environment following the intervention (5.9 to 39.8%, *p* < 0.01). Overall ABC compliance, including both sleep position/location and environment, improved from 4.4% pre-intervention to 32.5% post-intervention (*p* < 0.01). There was no significant variability between the hospitals (*p* = 0.71, *p* = 1.00).

**Conclusions:**

The AAP’s safe sleep recommendations are currently not upheld in children’s hospitals, but safer sleep was achieved across three children’s campuses in this study. Significant improvements were made in sleep environment and overall safe sleep compliance with this multi-pronged initiative.

## Background

The Centers for Disease Control and Prevention (CDC) defines sudden unexpected infant death (SUID) as “the sudden death of an infant under 1 year of age that cannot be explained after thorough investigation.” (Patton et al. 2015; Centers for Disease Control and Prevention 2019) SUID is routinely classified as: 1) sudden infant death syndrome (SIDS), 2) accidental suffocation and strangulation in bed (ASSB), or 3) death from unknown causes. Each year, approximately 4000 U.S. babies die from SUID. (Georgia Department of Public Health 2016) SUID is associated with unsafe sleep practices. (Erck Lambert et al. 2018; Moon 2016) Between 1990 and 1999, the SUID rate drastically declined following numerous safe sleep campaigns, of which the “Back to Sleep” campaign in 1994 was the most well-known. In 2012, the National Institute of Health (NIH) expanded their focus to include environmental recommendations (such as sleep location and environment) and renamed it the “Safe to Sleep” campaign. (Moon 2016; National Institute of Child Health and Human Development/National Institutes of Health Safe to Sleep Campaign 2019) Since 1997, SIDS deaths have become less common; however, infant death due to unknown causes and ASSB rates are stagnant. (Centers for Disease Control and Prevention 2019; Moon 2016; Shapiro-Mendoza 2017)

To reduce sleep deaths, the American Academy of Pediatrics’ (AAP) ABCs of sleep are paramount: place infants **A**lone, on their **B**ack and in a clear **C**rib with only a firm mattress and a tight fitting sheet. (Georgia Department of Public Health 2016) It has been shown caregivers are more likely to adopt a particular behavior if they are exposed to it in a trusted setting, such as a hospital. (Heitmann et al. 2017) However, hospitalized infants are often found in unsafe sleep environments. (Mason et al. 2013; Leong et al. 2019) Multiple studies have utilized crib audits to collect information such as patient age, sleep position/location and environment, followed by a quality improvement (QI) program in children’s hospitals and birthing centers; they found that safe sleep practices could be greatly improved. (Mason et al. 2013; Leong et al. 2019; Macklin et al. 2019; Shadman et al. 2016; Macklin et al. 2016; Moon 2011)

In response to Georgia’s high infant mortality rate, the Georgia Safe to Sleep campaign was initiated in 2016 to model and educate both parents and caregivers on safe sleep practices in mother-baby units at birthing hospitals. (Georgia Department of Public Health 2016) In 2017, we performed a study at a freestanding tertiary care children’s hospital to study the effectiveness of a quality initiative aimed at safe sleep compliance before and after QI interventions. (Leong et al. 2019) We hypothesized baseline compliance with safe sleep recommendations on the general pediatric inpatient units in all three hospitals would be poor, but that there would be significant improvement with a multipronged quality initiative. The objectives of this study mirrored our prior study, the difference being a larger sample size, a greater variety in patient population, and expansion to multiple campuses.

The objectives were:

1. To assess baseline infant sleep behaviors at three children’s hospital campuses
2. To evaluate the effectiveness of a multi-pronged quality improvement (QI) initiative that included nursing education, identification of nurse safe sleep champions, crib audits, crib cards with safe sleep checklists, and tracking boards in improving adherence to the ABCs of safe sleep at three children’s hospital campuses
3. To demonstrate a successful safe sleep initiative can be expanded throughout a hospital system

## Methods

### Study design

This study is a pre-intervention and post-intervention study designed to determine the efficacy of a QI program focused on improving safe sleep practices in the inpatient setting of 3 children’s hospitals. This study’s methodology is based on a previous study by Leong et al., with the addition of two hospital campuses. (Leong et al. 2019) We evaluated infant safe sleep practices on inpatient general pediatric floors of two tertiary care hospitals and one community hospital, for a total of five inpatient floors using a validated crib audit tool. (Leong et al. 2019) The interventions of the study included: 1) nursing education, 2) identification of nurse “safe sleep” champions, 3) crib cards, 4) crib audits, and 5) weekly reporting of data showing ABC compliance via tracking boards. This study was found to be exempt by the hospital’s institutional review board (IRB).

The general pediatric inpatient units of these hospitals were chosen due to a higher likelihood that they would represent the general pediatric population, in comparison to subspecialty units (e.g. mother/baby units). The crib audits were conducted on infants less than 12 months old in all three hospitals on the five general pediatric units. The pediatric units included in this QI program contained between 20 and 35 beds each, depending on the campus, with children ranging in age from 3 days to 21 years. Patients who were less than 12 months of age and asleep at the time of the crib audit were included in the study. Patients were excluded from the study if they were awake, intubated, had craniofacial anomalies, were requiring non-invasive positive pressure ventilation or high-flow nasal cannula, less than 32 weeks’ gestation, had incomplete data in the crib audit, a current admission to an intensive care unit, or required isolette or temperature support.

### Data collection

Data were collected using the Georgia Department of Public Health’s crib audit tool, which has been validated and utilized when evaluating the efficacy of the safe sleep programs at the birthing hospitals and in the previously performed study. (Leong et al. 2019) The crib audit tool was modified slightly to identify which campus and floor was audited. This tool is comprised of a checklist that keeps track of observations made in a patient’s room. Our primary outcomes included sleep position/location, sleep environment, and overall ABC compliance. ABC compliance was defined as both safe sleep position/location and safe crib environment. A non-random, convenience sampling method was used.

If the child was awake, the investigators returned to the room after completing the crib audits for other infants on the unit and attempted the audit again. Data collection occurred over a time period of approximately 2 months for pre-intervention and 2 to 4 months for post-intervention (depending on campus location). Patients who had been audited on a previous day were not excluded, as each day was a new opportunity to assess sleep environment and position/location choices. Post-intervention audits were collected on the same units as pre-intervention audits and using the same convenience sampling method.

The first assessment when doing the crib audits was determining if the infant was asleep or awake. If asleep, the crib audit was completed – assessing for position/location, sleep environment, and presence of caregiver. In order to be classified as having a safe sleep position/location, infants were required to be supine in a crib or held by an awake adult. Unsafe sleep position or location included prone positioning, sleeping in a caregiver’s bed, being held by a sleeping adult, or sleeping in a device that was not a crib or bassinet (such as a swing).

The second assessment made during crib audits was the sleep environment. In order to be classified as having a safe sleep environment, the infant had to be alone in the sleeping environment. The presence in the sleeping environment of any soft or hard toys, extra blankets, medical equipment not in use, diapers, washcloths, clothing, or pillows were marked it as unsafe. Pacifiers, medical equipment in use, and/or a single swaddling blanket/sleep sack in use were allowed in the safe sleep environments. During this study, due to current hospital policy, head-of-bed elevation was still considered safe, if the infant met the position/location and environmental requirements.

### Intervention

Once baseline safe sleep data were collected, the quality improvement initiative was implemented. Crib cards were made, and nurses were asked to place the cards on every crib for an infant under 12 months of age. The crib cards consisted of a checklist with the ABCs of safe sleep. Reminders were given during morning huddle. Safe sleep champions also encouraged nurses and staff to remove unsafe items. At the end of each week, tracking boards with baseline compliance on the unit to date were placed in each unit’s nursing break room. The tracking boards included information on how they had progressed since the previous week, the proportion of infants who were compliant with the AAP’s safe sleep recommendations, and the proportion of infants with correctly hung crib cards. These tracking boards were meant to act as encouragement as well as real-time feedback. Between 24 h and 7 days after the use of the crib cards and the tracking boards, post-intervention audits were collected. The sampling method and crib audit tool used in the pre-intervention time period were used again for post-intervention data collection. The post-intervention goal for ABC compliance was 25%. This goal was selected as a realistic target based on the pre-intervention ABC compliance at hospital 1.

### Statistical analysis

Data were collected using REDCap©, an online database. Descriptive frequencies were calculated (counts and percentages) to assess the safe sleep behaviors for both phases of the crib audits. Chi-square or Fisher’s exact test (when appropriate) was used to assess safe sleep behaviors for both the pre-intervention and post-intervention periods. Three pre- and post-intervention comparisons were conducted to evaluate: 1) the proportion of infants in safe sleep position/location, 2) the proportion of infants sleeping alone, and 3) the proportion of infants with overall ABC compliance (safe positioning and environment). Logistic regression was used for both crib environment and overall ABC compliance to estimate the effect of each hospital and time periods. Interactions between hospital and time period were tested*. Rv3.6.0* (Vienna, Austria) was used for statistical analysis and conducted using *R Core Team* (Core Team 2018); figures were produced using the package *ggplot2*. (Wickham 2009)

## Results

### Crib audits

**Fig. 1:**
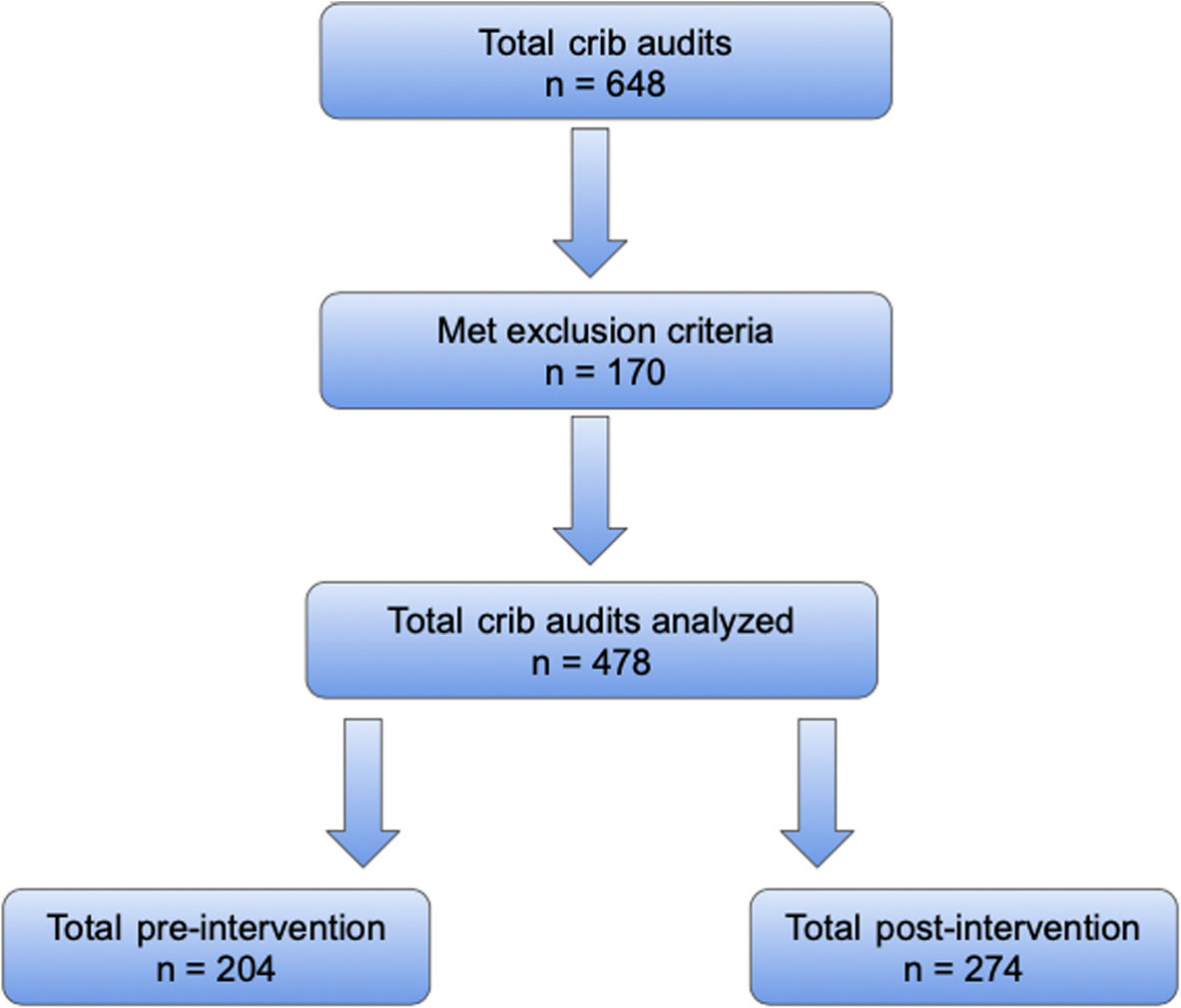
Patient inclusion flow chart

A total of 648 infants were screened among the three children’s hospital campuses. We excluded 170 infants. Thus, 478 infants were included for the final analysis, with 204 pre-intervention and 274 post-intervention (Fig. 1).

### Sleep position and location

A safe sleep position/location is defined as the infant on his/her back/supine or sleeping in the arms of an awake caregiver. Most infants both pre- and post-intervention were in a safe sleep position/location, 78.4% (95% CI 72.2–83.5%) and 76.6% (95% CI 71.3–81.3%) respectively (Table 1). There was no difference between pre- and post-intervention periods (*p* = 0.64). When analyzing unsafe sleep positions/locations prior to intervention, 3.9% were prone, 4.4% were sleeping on/in a caregiver’s bed, 3.4% were held by a sleeping adult, and 6.4% were side-sleeping. Following the intervention, 6.9% were prone, 7.3% were sleeping on/in a caregiver’s bed, 4% were held by a sleeping adult, and 4.4% were side-sleeping (Table 1).When safe sleep position/location is compared by hospital, utilizing logistic regression, there is no significant difference between hospitals (*p* = 0.36).

**Table 1:** Evaluation of sleep position/location* pre- and post-intervention

Variable	***n***	Pre (***n*** = 204)	Post (***n*** = 274)
Safe sleep position/location*, n (%)	478	n (%)	n (%)**
Not Safe		44 (21.6)	64 (23.4)
Safe		160 (78.4)	210 (76.6)
**Sleep position/location, n (%)**	478		
Sleeping on back in crib		131 (64.2)	162 (59.1)
Sleeping on side in crib		13 (6.4)	12 (4.4)
Sleeping held by sleeping adult		7 (3.4)	11 (4.0)
Sleeping in another device		1 (0.5)	0
Sleeping on stomach in crib		8 (3.9)	19 (6.9)
Sleeping in Crib with Sleeping Adult		6 (2.9)	2 (0.7)
Sleeping on/in caregiver’s bed		9 (4.4)	20 (7.3)
Sleeping held by an awake adult		29 (14.2)	48 (17.5)

*Safe Sleep Position/location: In order to be classified as having a safe sleep position/location, infants were required to be supine in a crib or held by an awake adult

**p = 0.64

### Sleep environment

The crib environments, when combining the results of all three campuses, were significantly safer post intervention, 5.9% (95% CI 3.3–10.1%) vs. 39.8% (95% CI 34.2–45.7%), (*p* < 0.01) (Table 2). The specifics of the items found in the cribs are listed in Table 2. There were statistically significant decreases in the following items found in the cribs post-intervention: clothing (22.1 to 6.6%, *p* < 0.01), diapers (21.6 to 4.7%, *p* < 0.01), stuffed toy (23.5 to 10.6%, *p* < 0.01), burp cloths (16.7 to 2.6%, *p* < 0.01), extra blankets (74 to 38.7%, *p* < 0.01), suction bulbs (5.4 to 0.4%, *p* < 0.01), fluffy blankets (27 to 17.5%, *p* = 0.02), wipes (28.4 to 9.1%, *p* < 0.01), plastic toys (9.3 to 3.6%, *p* = 0.01), and medical equipment not in use (27.5 to 5.8%, *p* < 0.01). There was no decrease in pillows and non-specified loose items found in the cribs post-intervention (Table 2). When comparing hospitals, adjusting for period in the logistic regression model, there are significant differences by intervention, but not by hospital. The test for interaction was not significant, meaning, the effect of the specific hospital on the crib environment does not differ by pre-intervention and post-intervention.

**Table 2:** Evaluation of crib environment^a^ pre- and post-intervention

Variable	Pre (***n*** = 204)	Post (***n*** = 274)	***p***-value
Environment, n (%)	n (%)	n (%)	< 0.01
Not safe	192 (94.1)	165 (60.2)	
Safe	12 (5.9)	109 (39.8)	
Clothing, n (%)	45 (22.1)	18 (6.6)	< 0.01
Diapers, n (%)	44 (21.6)	13 (4.7)	< 0.01
Stuffed toy, n (%)	48 (23.5)	29 (10.6)	< 0.01
Burping cloths, n (%)	34 (16.7)	7 (2.6)	< 0.01
Pillow, n (%)	27 (13.2)	22 (8.0)	0.07
Extra blanket, n (%)	151 (74.0)	106 (38.7)	< 0.01
Suction, n (%)	11 (5.4)	1 (0.4)	< 0.01
Fluffy blanket, n (%)	55 (27.0)	48 (17.5)	0.02
Wipes, n (%)	58 (28.4)	25 (9.1)	< 0.01
Plastic toy, n (%)	19 (9.3)	10 (3.6)	0.01
Bottle, n (%)	7 (3.4)	10 (3.6)	1.0
Med equipment NOT in use, n (%)	56 (27.5)	16 (5.8)	< 0.01
Electronics, n (%)	1 (0.5)	4 (1.5)	0.40
Bumpers, n (%)	0 (0.0)	2 (0.7)	0.51
Books, n (%)	1 (0.5)	0 (0.0)	0.43
Other loose items, n (%)	2 (1.0)	0 (0.0)	0.18

^a^Safe sleep environment defined as a clear crib except for pacifier and single swaddled blanket

### ABC compliance

**Fig. 2:**
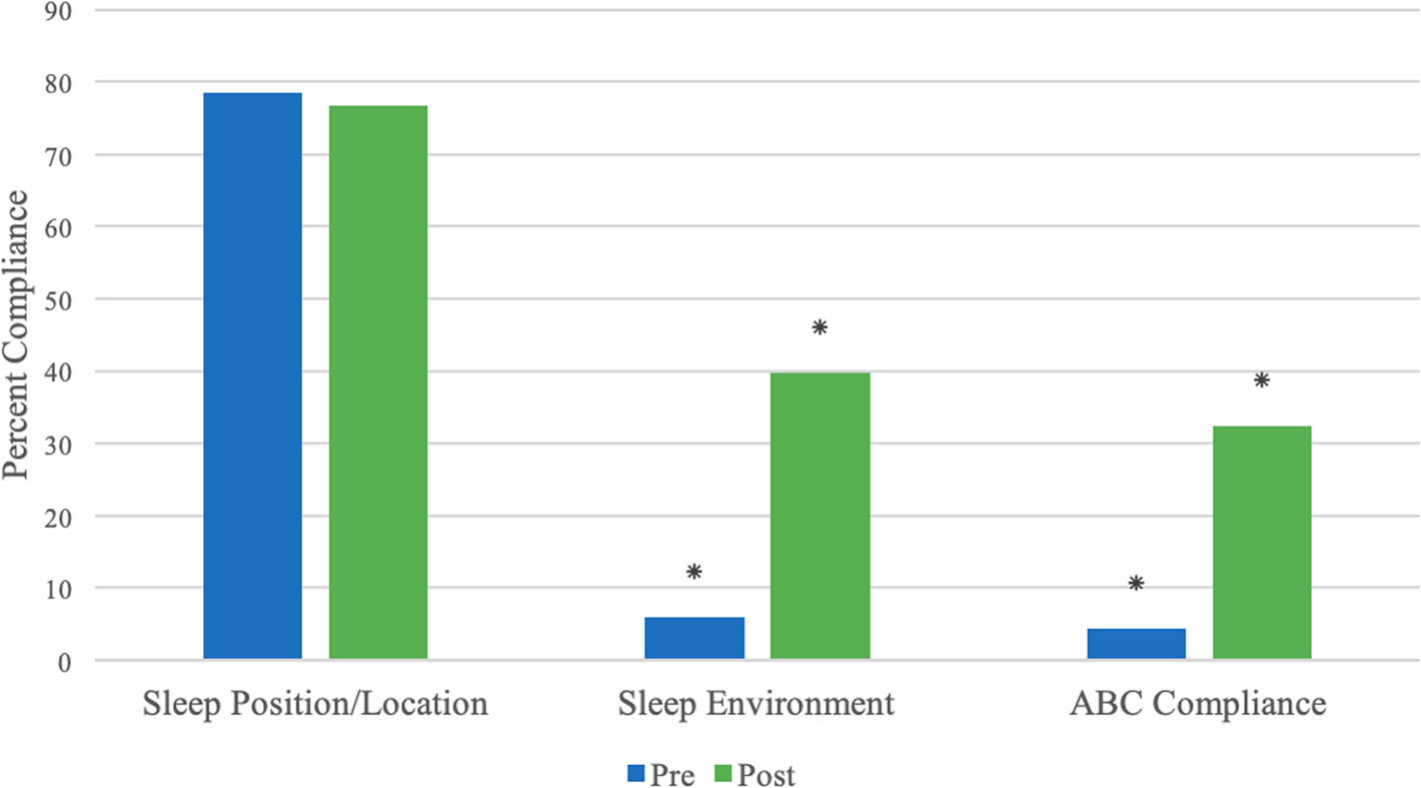
Pre- and post-intervention comparisons of sleep position/location, environment, and overall ABC compliance for all three campuses. *p < 0.01. *represents a significant improvement

ABC compliance (defined as including both safe sleep position/location and safe crib environment) significantly improved collectively among the three hospitals from 4.4% (2.0–8.2%) in the pre-intervention period to 32.5% (27.2–38.2%) to the post-intervention period (*p* < 0.01) (Table 3). ABC compliance improved, even after adjusting for each hospital (OR 10.6, 95% CI 5.4, 23.2). Of note, each campus met and exceeded the ABC compliance goal of 25% following the interventions. Figure 2 illustrates the changes found in all three categories: sleep position/location, sleep environment, and ABC compliance.

**Table 3:** Evaluation of ABC compliance pre- and post-intervention

Variable	Pre (***n*** = 204)	Post (***n*** = 274)	***p***-value
**ABC Compliance**^a^**, n (%)**	n, (%)	n, (%)	< 0.01
No	195 (95.6)	185 (67.5)	
Yes	9 (4.4)	89 (32.5)	

^a^ABC Compliance: compliant with both safe sleep position/location and environment

## Discussion

In this expansion of a safe infant sleep QI program, overall compliance with ABC recommendations improved significantly post-intervention. ABC compliance encompasses both sleep position/location and sleep environment, and among these two categories, a significant improvement was found only in sleep environment. As expected, and shown previously, sleep position/location did not improve to the same degree as sleep environment. (Leong et al. 2019) This is most likely due to the Back to Sleep campaign being well known and in place for many years leading to greater knowledge of what a safe sleep position and location entails. (Shapiro-Mendoza 2017; Kuhlmann et al. 2016; Colson et al. 2017; de Luca and Hinde 2016) Unfortunately, despite overall good compliance, there was still a moderate prevalence of unsafe positioning. Around 20% of infants were still found to be in unsafe sleep positions. Prior to the intervention, the most common unsafe sleep position was side sleeping. Following the intervention, the most common unsafe sleep position was on/in a caregiver’s bed.

Bed sharing has routinely been cited as a hazard to safe sleeping and the AAP recommends against it until 1 year of age, but especially in the newborn to six-month old age range. (Moon 2016) According to a 2015 study, 61% of caregivers report sharing a bed with their infant. (Centers for Disease Control and Prevention 2018) While several studies have linked it to an increased risk of SUID, it is still common practice. We hypothesize this may be seen especially in the hospital setting when caregiver’s feel the need to comfort their infant during a stressful time. In this study, the hospital policies allow for caregivers to sign a waiver allowing co-sleeping. In addition, this policy allows for replacing a crib with an adult-sized hospital bed, so the caregiver has a comfortable place to sleep with the infant. This is not standard hospital practice, and in the future, we hope to remove the co-sleeping waiver in order to discourage straying from the ABCs of safe sleep. It is clear additional education for caregivers on safe sleep is beneficial; however, it is unrealistic to expect caregivers to follow recommendations if healthcare professionals aren’t following them. Thus, it is important to understand the level of adherence to safe sleep recommendations in hospitals with the goal of improving safe sleep practices.

Following the implementation of the program, sleep environment was found to be significantly safer on the inpatient pediatric units with fewer items found in the cribs. Many of the items found in the cribs prior to the intervention were soft in nature - stuffed toys, diapers, burping cloths, pillows, fluffy blankets, extra blankets, and clothing. Loose, soft objects can obstruct airways and in doing so, increase the risk of SIDS and ASSB. (Moon 2016; Kanetake et al. 2003; Kemp et al. 1998; Patel et al. 2001; Shapiro-Mendoza et al. 2015; Scheers et al. 1998) The initial low compliance with a safe sleep environment supports the claim that the Back to Sleep campaign succeeded in increasing awareness about sleep position but did not result in universal adoption of best practices. It is clear more education and positive examples are needed. (Shadman et al. 2016; Macklin et al. 2016; Kuhlmann et al. 2016) Past studies suggest placing storage bins next to or at the end of cribs may be useful in encouraging the removal of nonessential items from the infant’s crib. (Kuhlmann et al. 2016; McMullen et al. 2016; Zachritz et al. 2016) Despite significant decreases in certain items in cribs, an increase in items such as bottles, electronics, and bumpers were noted. This is likely due to the small sample size that reported these items. A study with a larger sample size would be needed to further analyze these items. To improve safe sleep compliance in the future, the development and implementation of a computer-based training (CBT) program, currently in process, may be useful.

### Limitations

Data were collected from a convenience sample over a limited time period in a single state; however, 3 different hospitals were included. Because all interventions were performed at once, our statistics are reported as pre- and post-intervention results, as opposed to true PDSA cycles. Therefore, it is unclear which intervention had the greatest effect on compliance. If an infant was awake at the time of the audit, a second attempt was made to obtain the observational data. However, there was no required amount of time to wait between attempts, which may have led to a decrease in the potential sample size. Different reviewers performed audits and while the crib audit tool was made to be quite specific, different interpretations of certain items could have occurred. Crib card compliance was not consistently recorded in this study; therefore, it is difficult to determine the true impact of the crib cards. Another limitation of the study results is whether the interventions caused the significant increase in safe sleep or whether the Hawthorne Effect was heavily present. The changes in compliance found in this study may partly be the result of nurses and caregivers knowing they were being observed, and thus were more likely to follow safe sleep recommendations. We did not collect data on crib card compliance, so we cannot say that the compliance was high enough to combat the Hawthorne Effect.

## Conclusions

A safe infant sleep QI program at three children’s hospital campuses involving crib audits, nursing education, crib cards, and weekly tracking boards improved overall compliance with the ABCs of safe sleep. The program impacted sleep environment more than sleep position/location, which we believe is due to the long history of supine sleep recommendations. It is still unclear what specific element of the intervention caused the significant improvement in safe sleep, but the multi-pronged approach created a safer sleep environment for hospitalized infants. Efforts to increase compliance with well-known safe sleep recommendations in hospitals are needed, and long-term solutions such as storage bins and permanent safe sleep checklists may help.

## Data Availability

The datasets used and/or analyzed during the current study are available from the corresponding author on reasonable request.

## Abbreviations

AAP: American Academy of Pediatrics
ASSB: Accidental Suffocation and Strangulation in Bed
CBT: Computer Based Training
CDC: Centers for Disease Control and Prevention
IRB: Institutional Review Board
NIH: National Institutes of Health
QI: Quality Improvement
SIDS: Sudden Infant Death Syndrome
SUID: Sudden Unexpected Infant Death
U.S.: United States
